# Online Health-Seeking Behaviors and Information Needs Among Patients With Lymphoma in China: Study of Regional and Temporal Trends

**DOI:** 10.2196/80497

**Published:** 2025-11-18

**Authors:** Kaida Ning, Hongfei Gu, Meredith Franklin, Xiaoying Yang, Rong Wei, Zhen Song, Hong Xu, Ling Li Leng, Mengting Liu, Ju Dai, Jin Zhang, Rui Zeng, Yongshuai Hou, Rongjie Wang, Zirong Liu, Chenyang Huang, Runfa Cai, Huiling Liu, Li Charlie Xia

**Affiliations:** 1 Department of Network Intelligence Peng Cheng Laboratory Shenzhen, Guangdong China; 2 Department of Patient Services HOUSE086 Beijing China; 3 Department of Statistical Sciences University of Toronto Toronto, ON Canada; 4 Department of Statistics and Mathematical Finance South China University of Technology Guangzhou, Guangdong China; 5 Institute of Hematology Peking University People's Hospital Beijing China; 6 Department of Sociology Zhejiang University Hangzhou, Zhejiang China; 7 School of Biomedical Engineering Sun Yat-sen University Shenzhen, Guangdong China

**Keywords:** online patient forum, large language model, regional inequities, socioeconomic factors, health-seeking behavior, digital health, artificial intelligence, AI

## Abstract

**Background:**

Health disparities are closely associated with socioeconomic inequalities. Although this relationship is well recognized in the context of traditional health care access, its influence on online health-seeking behaviors such as posting questions on patient forums and seeking peer responses remains poorly understood, particularly in the context of resource-limited regions. Furthermore, it is unclear what types of questions are most frequently asked online and to what extent these questions receive helpful responses.

**Objective:**

This study aims to examine how socioeconomic status influences online health-seeking behavior by analyzing regional disparities in forum participation and their correlation with economic development. In addition, it aims to identify unmet informational needs among patients with lymphoma through large language model (LLM)–based forum thread classification and expert evaluation of forum responses by using data from the largest online blood cancer forum in China.

**Methods:**

We analyzed over 110,000 patient-initiated forum threads posted between 2012 and 2023, covering all the provinces of mainland China. Regional trends in forum participation rates were examined and correlated with economic development, as measured by gross regional product per capita. Second, an LLM was used to classify the threads into 6 predefined topics based on their semantic content, thereby providing an overview of the topics that users cared about. Additionally, an expert manual review was conducted based on relevance, accuracy, and comprehensiveness to assess whether users’ questions were adequately addressed within the forum discussions.

**Results:**

Regional forum participation rates were significantly associated with levels of regional economic development (Wilcoxon rank-sum test; *P*<.001), with the highest participation rates in the East Coast regions. Participation rates in less-developed regions steadily increased, reflecting the growing public demand for accessible health information. LLM-based analysis revealed that most discussions centered on medical concerns such as interpreting reports and selecting treatment plans across all regions. However, only 37% (117/316) of the user questions received useful responses, underscoring persistent gaps in access to reliable information.

**Conclusions:**

To our knowledge, this study represents the most comprehensive real-world investigation to date of spontaneous online forum participation and information needs among patients with cancer. Our findings highlight the necessity for government and health care providers to implement initiatives such as artificial intelligence–driven information platforms and region-specific health education campaigns to bridge information gaps, reduce regional disparities, and improve patient outcomes across China.

## Introduction

### Background

Health disparities are intertwined with socioeconomic inequalities [1,2]; however, the interplay between socioeconomic factors and online health-seeking behaviors such as posting threads on patient forums and seeking advice for symptoms or treatments remains poorly understood, particularly in developing nations. To address this gap, we leveraged China’s largest online forum for patients with blood cancer as a case study to explore this relationship and identify unmet informational needs among patients. This forum focuses on lymphoma, a major type of blood cancer. The treatment for lymphoma is often prolonged, typically lasting over a year, and may be extended in case of relapse [3,4]. Following discharge, patients face numerous challenges and require ongoing access to reliable health information. Therefore, knowledge about treatment options, managing side effects, and lifestyle adjustments is essential for patients to navigate the disease and improve their quality of life [5,6].

Over the past decade, the widespread adoption of the internet and the increasing accessibility of personal computers and smartphones have led a growing number of patients to seek health information and peer support through online forums. Among these, HOUSE086 [7], launched in 2011, is China’s largest blood cancer–specific forum. It serves as a platform for users to share their personal experiences, ask questions, and discuss a wide range of topics related to lymphoma. With over a decade of continuous operation and participation from users across the country, the forum has amassed a substantial body of user-generated content, offering valuable insights into the real-world concerns and informational needs of patients and their caregivers.

An in-depth analysis of online forum data provides valuable insights into regional disparities in health-seeking behaviors and patient information needs. A key focus of this investigation is the geographical distribution of users across regions with varying levels of economic development, measured by gross regional product (GRP). Individuals in more developed regions often benefit from better access to medical professionals, higher educational attainment, and greater openness to digital health tools, making them more likely to seek help online [1,8,9]. In contrast, users in less-developed regions, where health care access is limited, may rely more heavily on the internet for medical information and support [10,11]. These dynamics highlight the importance of exploring regional differences in online forum participation. Furthermore, the unique needs and concerns of patients with lymphoma across diverse regions remain underexplored. By leveraging large-scale real-world data, we can gain insights into patient experiences and effectively tailor digital health resources. Finally, it is worthwhile to study the extent to which user questions are adequately addressed within these forums—a critical factor in assessing the effectiveness of peer-to-peer support and information exchange among patients with cancer and their caregivers—and to help guide public policy and resources toward the areas of greatest need.

### Study Aims

Little is known about whether online patient health-seeking behavior is associated with regional economic development. At the same time, forum discussions often reveal questions and concerns that remain unmet by the formal health system, yet supporting evidence is limited. To address these issues, we analyzed more than 110,000 threads from China’s largest forum for patients with lymphoma to explore regional disparities in patient information-seeking behaviors, identify the topics that patients are interested in, and evaluate the responsiveness of online peer support.

## Methods

### Data for This Study

The forum data analyzed in this study were obtained from HOUSE086 [7], the largest online platform dedicated to patients with lymphoma in China. HOUSE086 is a peer-to-peer support community in which individuals affected by lymphoma, including patients, caregivers, and survivors, can share their experiences, exchange information, and seek advice. Upon registration, users agree that their personal information will remain private. However, any forum threads they submit are public and may be accessed by third parties, such as other users or researchers.

**Figure 1:**
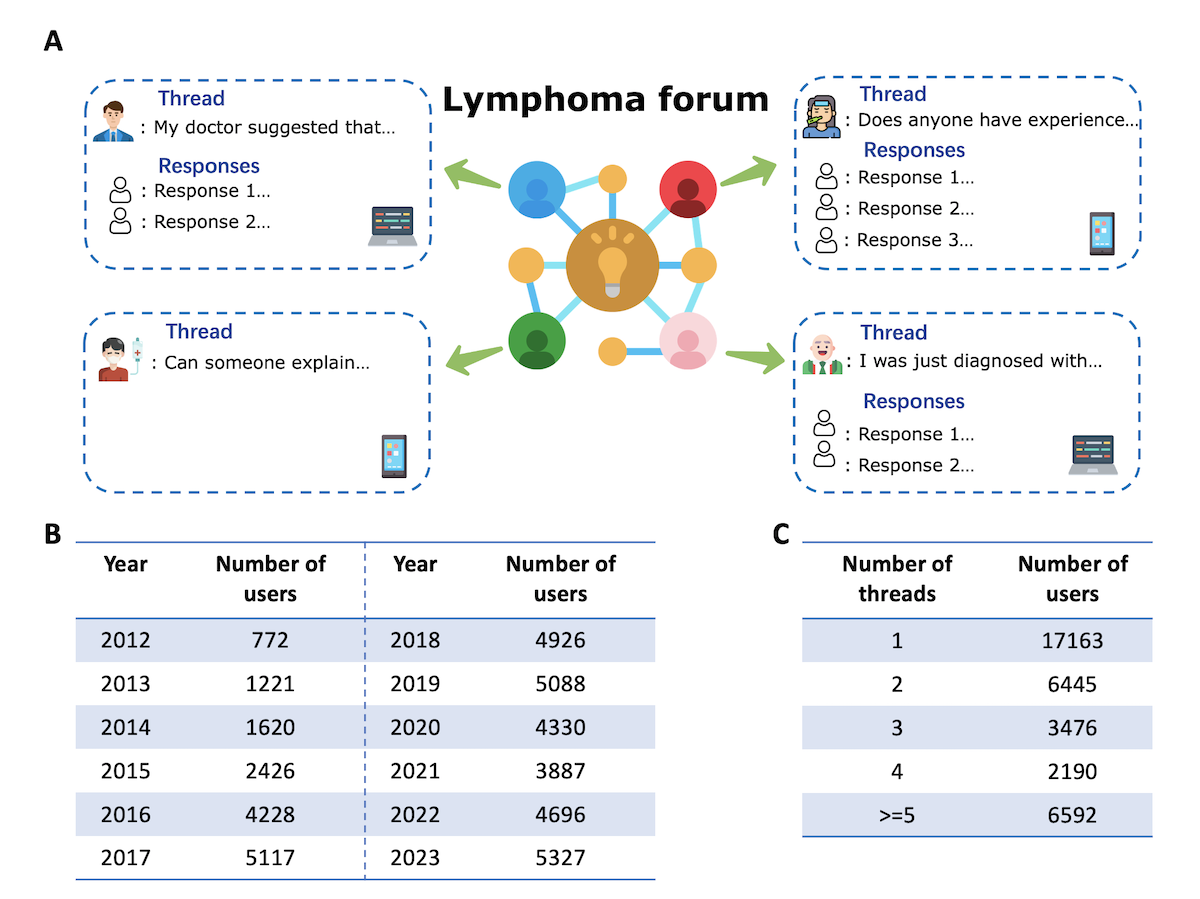
Data summary. (A) Illustration of user activities on the HOUSE086 forum, including example threads. In the forum, users can initiate and respond to threads spontaneously, and the number of responses to a thread is random. (B) Number of unique users who posted threads from 2012 to 2023. User participation rose steadily from 2012 to 2016, peaked at ~5000 annually in 2017-2019, dipped in 2020-2021, and rebounded to a record high in 2023. (C) Number of threads posted per user. Overall, 47.9% (17,163) of the users posted once, 18% (6445) twice, and 34.1% (12,258) three or more times.

On the forum, users initiate discussions by posting threads, which typically consist of a question or personal story. For example, users may seek specific advice on treatment: “In the case of follicular recurrence, should we adopt a watchful waiting approach or proceed with immediate treatment?” or seek moral support through a comment about a loved one: “My mother has developed secondary central nervous system lymphoma, and I am really scared right now.” Refer to Figure 1A and Multimedia Appendix 1 for more details (examples of forum threads presented in this manuscript were translated from simplified Chinese to English using ChatGLM [12,13]). These threads are open to responses from other community members. In total, data on 118,542 threads posted in the patient peer support panel were collected. Each thread included a title and content (Multimedia Appendix 1), along with a timestamp and the user’s IP address.

**Figure 2:**
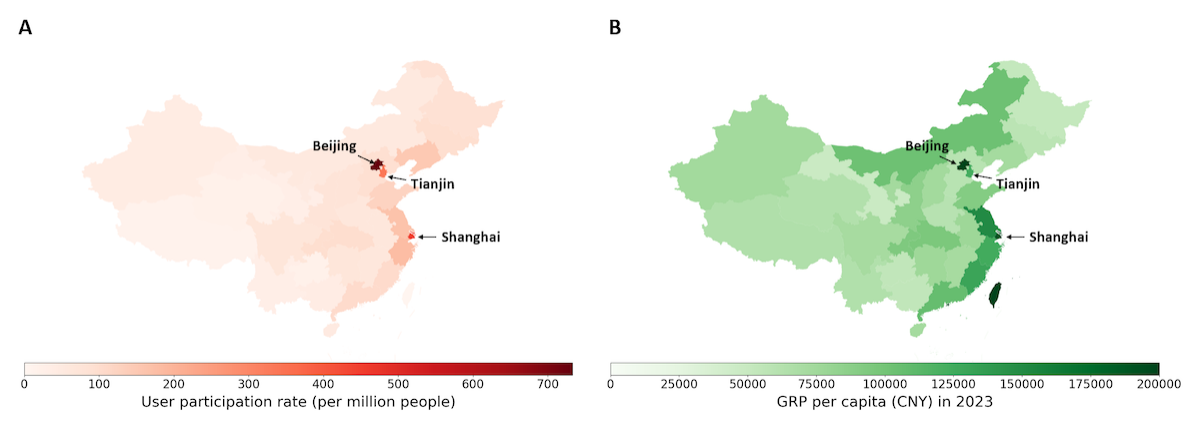
Regional forum user participation rate is significantly correlated with GRP per capita across administrative regions 2023 (ρ=0.87; *P*<.001). (A) Number of forum users who posted, grouped by administrative regions in China and normalized by regional population size (aggregated data from all years). (B) GRP per capita for each administrative region in China in 2023. GRP: gross regional product.

Data on population size and GRP for all administrative regions in China were obtained from government websites specific to mainland China [14], Taiwan [15], Macau [16], and Hong Kong [17]. We observed a very low participation rate from Taiwan, Macau, and Hong Kong. One possible reason is that these regions, outside of mainland China, predominantly use traditional Chinese and may not be familiar with simplified Chinese, whereas HOUSE086 uses simplified Chinese. Another reason may be the limited awareness of HOUSE086 in these regions. Accordingly, we included statistics for Taiwan, Macau, and Hong Kong only in Figures 1 and 2 but restricted the remaining analyses to mainland China. We focused on mainland China mainly because the forum participation rate from Taiwan, Macau, and Hong Kong was very low, and given their high GRP, our central finding—that patient participation was higher in regions with higher GRP—would not meaningfully apply to these regions.

### Using a Large Language Model to Classify Threads Into Topics

We used semantic analysis to classify the threads into specific categories with ChatGLM, a large language model (LLM) trained with extensive Chinese content [12,13]. We selected ChatGLM for two main reasons: (1) it is readily accessible within mainland China, thereby avoiding cross-border data transfer, and (2) it has demonstrated performance comparable to GPT-4 in Chinese-language tasks [18]. We input the thread into ChatGLM and used zero-shot prompting [19,20], where the LLM was instructed to classify a thread into 1 of 6 predefined topics, without any model fine-tuning on this classification task. The topics were determined by a clinician and a layperson jointly through a manual evaluation of 418 threads posted on July 1 of each year (this date falls in the middle of the year and does not coincide with major Chinese holidays). The six topics were as follows: (1) interpretation of medical reports, (2) treatment plan selection, (3) general medical-related questions, (4) expressing feelings, (5) treatment cost questions, and (6) other topics. If a thread was classified into “general medical-related questions” in the first round, we used ChatGLM one more time to further classify these threads into 4 predetermined subcategories. Multimedia Appendix 1 includes Python code for zero-shot prompting and an example showing how a thread was entered into ChatGLM as a prompt to obtain topic classification results. In the task for classifying the 418 threads into 6 topics, the agreement between zero-shot classification and manual classification was 86%, with a Cohen κ of 0.82, indicating good interrater reliability and validating the model’s utility for larger-scale analysis.

### Evaluating Responses to Threads

To evaluate the responses to threads, we examined those posted on July 1 of each year from 2012 to 2023. The purpose was to assess whether user-initiated threads containing questions received helpful responses from fellow forum participants. These threads and their corresponding responses were reviewed by a hematologist who evaluated the responses for their relevance, accuracy, and comprehensiveness.

### Prompts and Statistical Analyses

**Figure 3:**
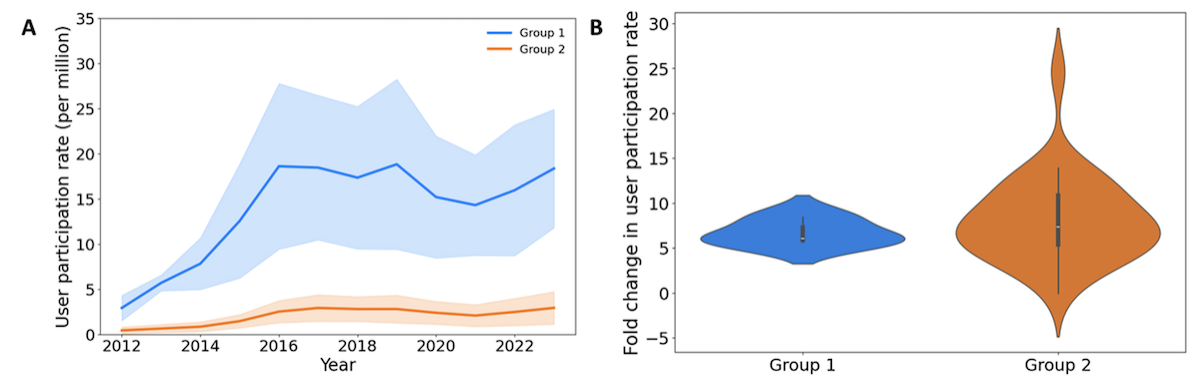
Comparison of user growth between group 1 (Beijing, Shanghai, and Tianjin) and group 2 (all other regions). (A) Group 1 regions exhibited a higher absolute growth in user participation rate compared to group 2 regions. (B) No significant difference was observed in the fold change of user participation rate between the two groups.

All data exploration and the prompts for using ChatGLM in thread topic classification were conducted in Python. Statistical analyses, including the Wilcoxon rank-sum test and Cohen κ statistic, were also performed in Python. We observed that the distribution of group 2 was skewed (violin plot in Figure 3B). Therefore, we selected the nonparametric Wilcoxon rank-sum test for group comparison.

### Ethical Considerations

This study analyzed publicly available, deidentified data from the HOUSE086 patient forum. All forum contents are publicly accessible, and users agree upon registration that their contributions may be viewed by others and used for research purposes, whereas personal information remains private. The platform’s terms of use permit analysis of the forum content without further consent. Therefore, no additional informed consent was required. All figures and analyses present only aggregated or anonymized results. This study was exempt from ethical review. In accordance with Article 32 of the Measures for Ethical Review of Life Sciences and Medical Research Involving Humans (National Health Commission, Ministry of Education, Ministry of Science and Technology, and National Administration of Traditional Chinese Medicine, 2023), studies using publicly available, legally obtained, and deidentified data that do not harm individuals or involve sensitive personal information are exempt from ethical review [21].

## Results

### Summary of Data

A total of 118,542 threads were posted by 35,866 HOUSE086 users between 2012 and 2023. In 2012, fewer than 1000 forum users initiated the threads. This number increased steadily from 2012 to 2016 and peaked between 2017 and 2019, during which time around 5000 users posted each year. Following a modest decline from 2020 to 2021 (likely due to the impact of COVID-19), user participation rebounded from 2022 to 2023, reaching an all-time peak in 2023 (Figure 1B). Over the study period, a large portion of users posted only once: 47.85% (17,163/35,866) were single-time contributors, 17.97% (6445/35,866) posted twice, and 34.18% (12,258/35,866) posted 3 or more times. On average, users contributed 3.3 threads each (Figure 1C).

### Geographical Trends in User Participation

We found substantial regional variation in forum participation rates across China. Participation rate was defined as the number of forum users from a given region normalized by the total population of that region. Overall, East Coast regions exhibited higher participation rates compared with inland regions (Figure 2). These coastal areas tend to have a higher GRP per capita. A statistically significant positive correlation was observed between the regional participation rate and per capita GRP in 2023 (ρ=0.87, *P*<.001). Notably, this positive correlation was consistent across all years from 2012 to 2023, with Spearman correlation coefficients exceeding 0.78, with *P*<.001 (Figures S1-S2 and Table S1 in Multimedia Appendix 1).

Among the East Coast regions, Beijing, Shanghai, and Tianjin (BST) had the highest participation rates. A previous study found that these regions have the highest level of Human Development Index in China [1]. To further test regional differences, we created 2 administrative groups: group 1, consisting of BST; and group 2, comprising all other regions. Forum participation in group 1 had a notably higher absolute growth in the participation rate than that in group 2 (Figure 3A). However, the fold change in participation rates did not differ significantly between the groups (Wilcoxon rank-sum test, *P*=.60; Figure 3B).

### Qualitative Analysis of Threads

To gain an overview of thread content, we used an LLM, ChatGLM, to categorize each thread into 1 of 6 independent categories (see the Methods section for details). Majority of topics were medical-related inquiries, including (1) interpretation of medical reports or examination results (41,159/118,542, 34.72%), (2) selection of treatment plans (23,849/118,542, 20.12%), and (3) general medical-related questions (36,017/118,542, 30.38%), (4) statements expressing feelings (4517/118,542, 3.81%), (5) inquiries about treatment costs (2562/118,542, 2.16%), and (6) unclassified topics (10,438/118,542, 8.81%). The larger category 3, general medical-related questions, was further subdivided into 4 subtopics: drug side effects and treatment-related comorbidities (20,950/36,017, 58.17%), recommendations for hospitals and doctors (8377/36,017, 23.26%), explanations of medical terms (3956/36,017, 10.98%), and channels for purchasing medication (2734/36,017, 7.59%). The details are illustrated in Table 1.

**Figure 4:**
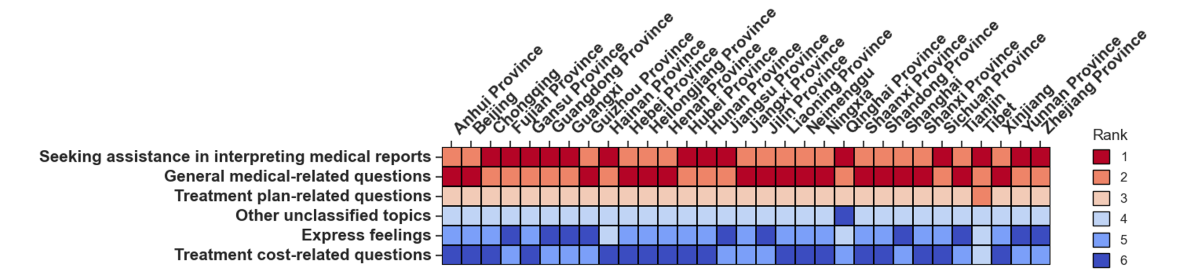
Ranking of thread topics across administrative regions: the values within each cell indicate the rank of a given topic based on its popularity in that region (with 1 indicating the most frequent). For example, in Anhui Province, "General medical-related questions" ranks first, while "Seeking assistance in interpreting medical reports" ranks second.

Although participation rates varied geographically, medical-related questions predominated across all regions (Figure 4). Notably, general medical-related questions were among the most frequent from 2012 to 2017. Starting in 2018, questions on interpreting medical reports gradually became the most common in most regions (Figure S3 in Multimedia Appendix 1).

After characterizing the categories of threads raised on the forum, we examined the extent to which threads involving questions were addressed by other forum users. There were in total 316 threads with user-raised questions that were posted on July 1 of each year between 2012 and 2023. A total of 304 (96.2%) questions received follow-up responses. However, a hematologist conducted a manual review and found that only 117 (37.0%) of these questions received useful insights from the responses (see the Methods section for details).

**Table 1:** Distribution of user-raised thread topics (aggregated data from all the years; N=118,542).

Topic, subtopic			Values, n (%)
Seeking assistance in interpreting medical reports			41,159 (34.72)
Treatment plan selection questions			23,849 (20.12)
**General medical-related questions**			36,017 (30.38)
	Drug side effects and treatment-related comorbidities^a^	20,950 (58.17)	
	Requesting recommendations for hospitals and doctors^a^	8377 (23.26)	
	Explanation of medical terms^a^	3956 (10.98)	
	Channels for purchasing medication^a^	2734 (7.59)	
Expressing feelings			4517 (3.81)
Treatment cost–related questions			2562 (2.16)
Other topics			10,438 (8.81)

^a^In these cases, the percentages were calculated over n=36,017.

## Discussion

### Principal Findings

To our knowledge, this study represents the largest real-world analysis to date on online health-seeking activities and discussion topics among patients with cancer in China, and possibly the largest of its kind globally. By analyzing over 110,000 threads posted by users on the HOUSE086 forum from 2012 to 2023, we gained insights into the online participation patterns of patients and families over time and across different regions. Furthermore, we used zero-shot learning with LLM to comprehend the information needs of patients.

The number of users who posted threads on the forum for patients with lymphoma increased steadily from 2012 to 2016, peaking between 2017 and 2019. After a slight drop from 2020 to 2021 (likely due to the COVID-19 pandemic), user participation recovered from 2022 to 2023, reaching a peak level. The deceleration in growth post-2020 can be attributed to the proliferation of internet-based health care platforms in China such as Haodaifu, Health 360, and Ping An Health, as well as the burgeoning popularity of WeChat patient groups, from where patients and their families are increasingly seeking assistance and support [22-24].

Our analysis revealed a persistent imbalance in forum participation, with users from more economically developed regions contributing at disproportionately higher rates. Participation rates in the East Coast regions, particularly in BST, were higher than those in inland regions (Figure 2). This trend has remained consistent over the past decade. Furthermore, from 2012 to 2023, BST showed a greater increase in participation than other regions. This disparity is likely due to uneven economic development and infrastructure investment, resulting in a concentration of high-quality hospitals in the East Coast regions [25,26]. BST hosts many of the country’s top-tier medical facilities, making diagnosis and treatment easier. These cities also attract many patients from other regions, who often post questions online after consultations. Furthermore, patients and their families in these regions tend to be better educated and more aware of the benefits of seeking help online [1,8,9]. By contrast, it is also worth noting that, when considering the fold change in the participation rate from 2012 to 2023, there was no significant difference between BST and the other regions (Figure 3). This was correlated with the rising accessibility of personal computers and mobile phones, which facilitated broader access to online communities for individuals across the country [27]. Moreover, in less-developed regions, the relative scarcity of local hematological cancer specialists heightened the residents’ motivation to seek support and information from these online communities [10].

To better understand the questions that patients with lymphoma have outside of hospital settings, we used zero-shot learning with an LLM to classify forum threads into topics. This approach allowed us to extract meaningful themes from large-scale patient-generated content, following similar methodologies recently demonstrated in studies of health-related texts [28,29]. We observed that most threads posted were medical in nature (ie, interpretation of medical reports or exam results, selection of treatment plans, and general medical-related questions). When analyzing topics by administrative regions and over the years, we observed a consistent trend among all regions. We found that from 2012 to 2017, topic 3 (general medical questions) was among the most frequently asked questions. Starting from 2018, topic 1 (medical report interpretation) gradually became the most frequently asked question in most regions. This shift suggests that patients are increasingly attentive to their health details, seeking help to interpret specific clinical findings recorded in their medical reports.

It was reported that patients frequently turn to online forums to bridge gaps in clinical communication and seek peer support, particularly when navigating complex diagnoses, such as cancer [30]. However, qualitative analysis revealed that responses to questions raised by forum users were often limited and sparse, reflecting the forum’s peer-driven nature. For instance, a user might ask a treatment-related question only to receive responses that simply echo the concern without substantive guidance. We estimated that only 37% (117/316) of the questions received helpful responses. The lack of timely and informative feedback likely discouraged continued participation, as most users posted fewer than 3 times.

**Figure 5:**
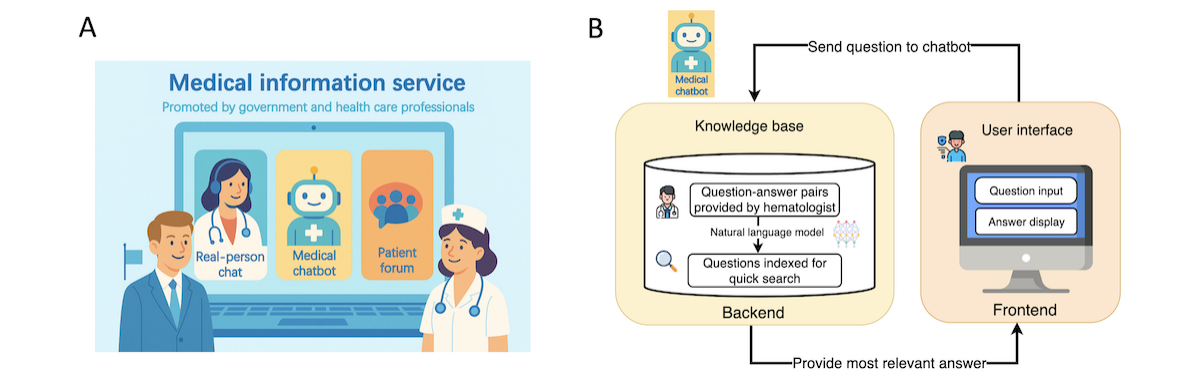
A proposed design of online medical information service promoted by government and health care professionals. (A) Online medical information service comprising real-person interaction, a medical chatbot, along with an online patient forum. (B) Design of a medical chatbot for information delivery. The backend consists of a knowledge base built from hematologist-provided question–answer pairs. A natural language model encodes both the knowledge base questions and user queries into vectors to enable efficient semantic retrieval.

Given the large number of lymphoma forum users seeking help online and the lack of timely responses to their questions, establishing a reliable online service for patients with lymphoma—and potentially for those affected by other diseases—is essential. Previous studies have emphasized the positive impact of online peer support groups that are moderated by hospital or clinic staff and trained mentors to supplement the needs of patients with cancer [31]. Our study suggests that establishing and promoting online medical information services by the government and health care professionals—including real-person interactions, medical chatbots, and online patient forums—is needed to address patients’ out-of-hospital information needs (Figure 5A). In China, emerging commercial internet hospitals now enable human doctors to answer patients’ questions online, which is a step in this direction. Furthermore, a chatbot with expert knowledge (also referred to as an FAQ system) could benefit patients with lymphoma and their families by providing quick answers to general medical-related questions, such as managing side effects and finding health care providers [32,33]. Recent studies have highlighted both the potential and the limitations of health chatbots. Habicht et al [34] deployed a personalized AI-enabled referral chatbot and reported that the tool significantly increased total referrals and improved access for underserved groups. In contrast, Phiri and Munoriyarwa [35] reviewed the use of 12 health chatbots in Africa and revealed insights into their popularity and accessibility but concluded that data collected at that time were insufficient to show whether chatbots were effectively supporting health in the region. Minimal maintenance and low supervision are required for the chatbot once it is established. It uses expert knowledge in the form of pre-matched question-and-answer sets and allows users to quickly obtain answers and determine the relevance of the information provided (Figure 5B illustrates the design of a medical chatbot). Moreover, mobile phone penetration has continued to grow in the inland regions of China. For example, as reported by People, a well-known Chinese news portal, the mobile phone penetration rate in Tibet reached 89.51 per 100 inhabitants by the end of 2022 [36]. This suggests that promoting mobile phone–based chatbots in inland regions is feasible at a relatively low cost. We acknowledge that although chatbots may provide a low-cost means of delivering health information, their effectiveness in addressing personalized medical information needs to be validated with real-world data. Furthermore, feasibility challenges remain, including the need for high-quality information sources for the chatbot, as well as effective promotion and sustained user participation, particularly in resource-limited regions.

Our study has some limitations. First, we focused on data from a forum for lymphoma patients in mainland China, which, to the best of our knowledge, is currently the largest of its kind. We did not conduct a systematic review of patient forums in Taiwan, Macau, and Hong Kong, where region-specific online platforms may better capture local health information needs. Furthermore, there are global forums for patients with lymphoma (eg, Lymphoma.com Community Message Forums, Leukemia and Lymphoma Society), and it would be valuable to compare our results with future analyses of data from these platforms. Second, there is a potential bias in IP-based regional assignments: IP location can only approximate the user’s city or region and may at times reflect the location of an operator’s data center rather than the user’s actual location. Furthermore, patients may travel to large cities. such as BST, to seek medical care and post questions shortly after a doctor’s visit. This could have led to a higher concentration of forum participation in these cities. Third, we used ChatGLM, an LLM trained on extensive Chinese-language content, to assist in the analysis of patient-initiated threads. In our analysis, the topic classification by ChatGLM aligns with that of a human investigator in most cases (ie, the agreement between zero-shot classification and manual classification was 86%). However, we acknowledge the potential for discrepancies between the 2 approaches, as mentioned in previous studies [37,38]. Resolving such discrepancies will provide a clearer picture of the distribution of topics in which forum users are interested. Fourth, we only assessed threads from a single date (July 1 of each year) for topic classification and response evaluation. Although this date was deliberately chosen to reduce confounding from holiday effects, it may not fully represent out-of-hospital information needs at other times. Seasonal factors, changes in health-seeking behaviors across the year, or temporal variations in online activities could influence both participation and content. Future research would benefit from sampling multiple dates or continuous data across the year to minimize temporal bias. Fifth, there are emerging commercial services that now offer physicians opportunities to answer patients’ questions online. The data collected by these platforms are highly valuable. However, we did not include these data in our analysis because they are typically proprietary and unavailable for research. Finally, in our study, we did not resolve disagreements in the classification results between LLM and human reviewers. A potential solution would be to identify categories with frequent discrepancies and address them by refining zero-shot prompts to better capture human rationale or by fine-tuning the LLM parameters with additional human-labeled examples.

### Conclusions and Implications

In conclusion, we conducted the largest real-world study to date on online health-seeking activities and informational needs among patients with lymphoma in China. We observed clear regional disparities in forum participation, which were closely associated with differences in economic development. Despite geographical variations, the types of questions raised by users were generally consistent across regions, with a strong focus on medical concerns. A critical issue identified was the lack of effective responses to user queries, highlighting the need for more structured and reliable support. To address these challenges, we recommend the establishment of services supported by the government and clinicians to provide accessible, high-quality online health information and improve equitable access nationwide. Our findings have broader implications, offering insights for other countries—particularly low- and middle-income regions—where online health information services may complement formal health care systems.

## Data Availability

The datasets generated or analyzed during this study are available from the corresponding author on reasonable request.

## Appendix

Multimedia Appendix 1 Examples of forum threads that were translated from simplified Chinese to English using ChatGLM and user participation data.

## Abbreviations

BST: Beijing, Shanghai, and Tianjin
GRP: gross regional product
LLM: large language model
