# Supplementary material for: Online Health-Seeking Behaviors and Information Needs Among Patients With Lymphoma in China: Study of Regional and Temporal Trends
Source: J Med Internet Res. 2025 Nov 18;27:e80497. doi: 10.2196/80497 (PMC12673299; doi:10.2196/80497)

**Supplemental Information**

**Section 1. Examples of threads**

We combine “title” and “content” posted by one user into a “thread”. Below are two examples of threads:

**Thread example 1**

Original webpage: <https://www.house086.com/thread-317404-1-1.html>

**Title:** In the case of follicular recurrence, should we continue watchful waiting or proceed with immediate treatment?

**Content:**

Hello everyone, my mother is 61 years old. She was first diagnosed with follicular lymphoma in October 2018, with bone marrow involvement. She received the R-CHOP regimen every 21 days for a total of 6 cycles, followed by two years of maintenance therapy with rituximab alone, without any oral medication. In July 2021, she completed treatment and achieved PR. After that, she had check-ups every six months. In March 2022, a new lymph node about 1 cm was found in her abdomen. In September 2023, the abdominal lymph node measured about 2 cm. In September 2024, an abdominal ultrasound showed 3 or 4 lymph nodes, measuring 5.3 cm × 2.2 cm and 3.3 cm × 1.9 cm. A biopsy of the abdomen confirmed it was still follicular lymphoma. The local hospital said it was a relapse and recommended hospitalization for treatment, but because my mother had no symptoms at the time, we chose to wait and see, considering her age and our concern that overly aggressive treatment might weaken her further. In March of this year, another examination showed about 3 or 4 abdominal lymph nodes measuring 5.5 cm × 2.2 cm and 3.9 cm × 1.9 cm, and an enhanced CT showed a short axis of about 2.7 cm. Compared with last September, there hasn’t been much change. Apart from her abdomen, she has no significantly enlarged lymph nodes elsewhere. However, the local hospital still advises hospitalization for treatment. The recommended regimens are BR or R-CHOP, or participation in a trial group (rituximab alone plus oral lenalidomide plus an experimental drug). Currently, my mother has no symptoms—no night sweats, no weight loss, and no itching. Should we continue to wait or proceed with hospitalization? I’m considering going to Beijing to consult a doctor about whether we should start treatment soon or continue to wait. However, we haven’t done a PET scan in nearly two years, and the pathology is from last September. Can I still bring the information I have now for consultation? Thank you in advance for your guidance and advice!

**Thread example 2**

Original webpage: <https://www.house086.com/thread-317371-1-1.html>

**Title:** My mother has indeed developed secondary central nervous system lymphoma, and I’m really scared right now.

**Content:** The data I saw online is truly frightening. Today, I spoke with the doctor and learned we might need to proceed with CAR-T therapy. When discussing this with my dad, I felt completely torn. Selling our house could cover the cost of commercial CAR-T treatment, but I'm terrified Mom would refuse. Moreover, the probability of recurrence still seems alarmingly high. Yet not pursuing treatment fills me with dread that we'll live with regret and endless "what-ifs". Most heartbreakingly, Mom is only 43 - so young - and I can't comprehend why her condition is progressing this rapidly. This overwhelming fear has me paralyzed.

**Section 2. Using ChatGLM to classify a thread**

Below is a running example of Python code we used to call ChatGLM for classifying threads into six pre-defined topics:

pip install zhipuai *# Install tools for using the ChatGLM model*

*from* zhipuai *import* ZhipuAI

*import* pandas *as* pd

*import* time

*# ChatGLM API key*

client = ZhipuAI(*api_key*="your key")*# A key for ChatGLM can be applied from https://bigmodel.cn/*

*# Load Excel file*

df = pd.read_excel("~/Downloads/my_data.xlsx") *#Load the data*

contents = df['Thread Content']

*# Prompt setting*

def build_prompt(*thread_content*):

*return* f"""#Context: The data comes from user threads in an online lymphoma patient community, your task is to classify these contents

# Task: Classify the following user question and output the name of the classification category (please only choose from the following six categories, and only return "Interpretation of test results","Questions related to treatment plan selection","Other treatment-related issues","Patient emotional support","Financial burden", or "Other"). If the thread content could belong to both "Interpretation of test results" and "Questions related to treatment plan selection", please prioritize classifying it as "Interpretation of test results". No explanation is needed.

# Categories include:

1. Interpretation of test results: Requesting help to review and interpret test results.

2. Questions related to treatment plan selection.

3. Other treatment-related issues (excluding treatment plan selection): For example, requests for recommendations of hospitals and doctors, side effects of drugs or bone marrow transplants and related complications, drug purchase channels, explanations of medical terms.

4. Patient emotional support: Expressing the emotional distress of patients and their families and the need for psychological support.

5. Financial burden: Mentioning treatment costs, medical insurance reimbursement, and other financial issues.

6. Other.

User thread content is as follows:

\"\"\"{thread_content}\"\"\"

Please return the name of the classification category for this thread"""

*# Save results*

results = []

*# call API*

*for* i, content *in* enumerate(contents):

prompt = build_prompt(content)

*try*:

response = client.chat.completions.create(

*model*="glm-4-air", *#specify the model type*

*messages*=[{"role": "user", "content": prompt}]

)

category = response.choices[0].message.content.strip()

results.append(category)

print(f"{i+1}/{len(contents)}: SUCCESS → {category}")

time.sleep(1.2)

*except* Exception *as* e:

print(f"ERROR {i+1}: {e}")

results.append("ERROR")

*# Save the results into an Excel file*

df['results'] = results

df.to_excel('~/Downloads/classified_output.xlsx', *index*=False)

print("✅ DONE 'classified_output.xlsx'")

Below is an example of a prompt for ChatGLM to classify a thread posted by a user, where ChatGLM responded with 'Questions related to treatment plan selection' as the classification result:

I am looking for hospitals that are good at traditional Chinese medicine treatments. I live in Henan Province and have follicular lymphoma. I have completed 6 cycles of R-CHOP treatment and the treatment has now ended. I would like to take some traditional Chinese medicine to improve my body's condition and boost my immune system. Does anyone know of any reliable hospitals?

**Section 3. Data availability**

**Data available:** Upon Request

**Additional information:** The data analyzed in this study is publicly accessible on the HOUSE086 forum (<https://www.house086.com/>). Structured data may be shared upon reasonable request through contacting the corresponding author.

**Section 4. Supplementary display items**


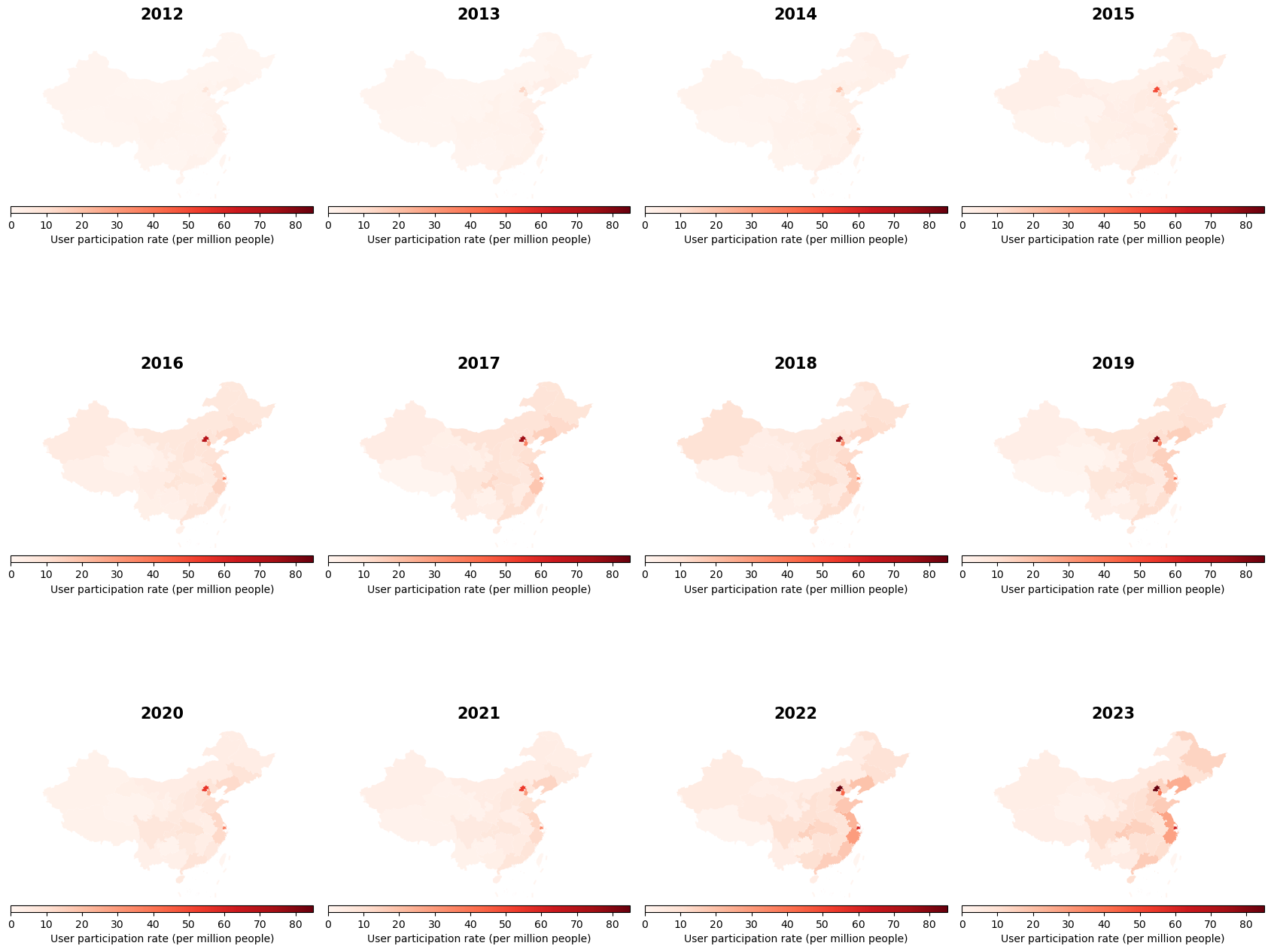


Figure S1. Number of forum users who posted each year, grouped by administrative divisions in China and normalized by the population size of each region, from 2012 to 2023.


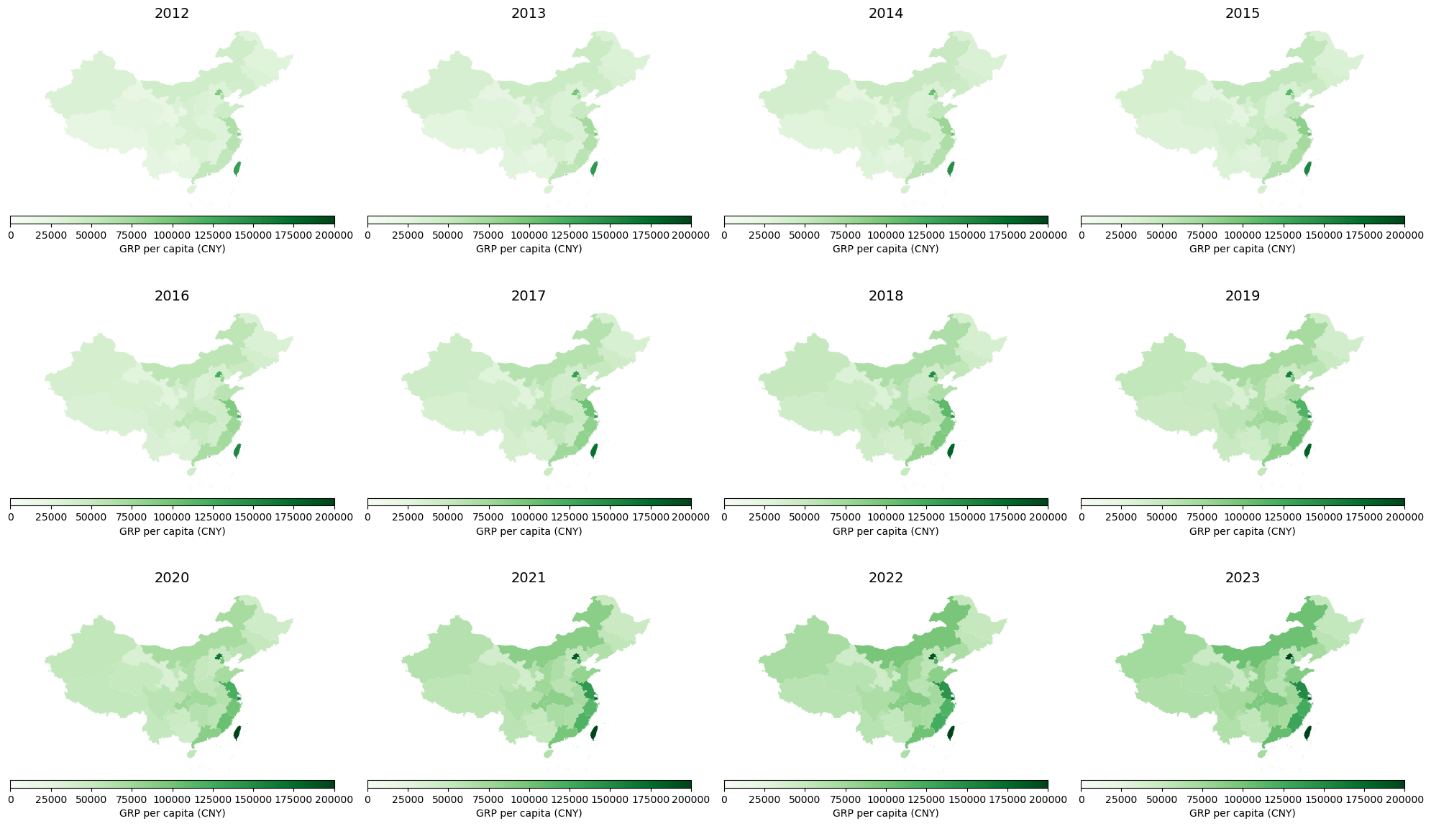


Figure S2. GRP per capita for each administrative region in China, from 2012 to 2023.

Table S1. Spearman correlation coefficient between regional participation rate and GRP per-capita, from 2012 to 2023.

| **Year** | **Correlation coefficient** | **P-value** |
| --- | --- | --- |
| 2012 | 0.87 | <.001 |
| 2013  2014  2015  2016  2017  2018  2019  2020  2021  2022  2023 | 0.80  0.78  0.83  0.84  0.89  0.87  0.85  0.84  0.84  0.87  0.86 | <.001  <.001  <.001  <.001  <.001  <.001  <.001  <.001  <.001  <.001  <.001 |

Figure S3. Distribution of thread topics across administrative regions, from 2012 to 2023.


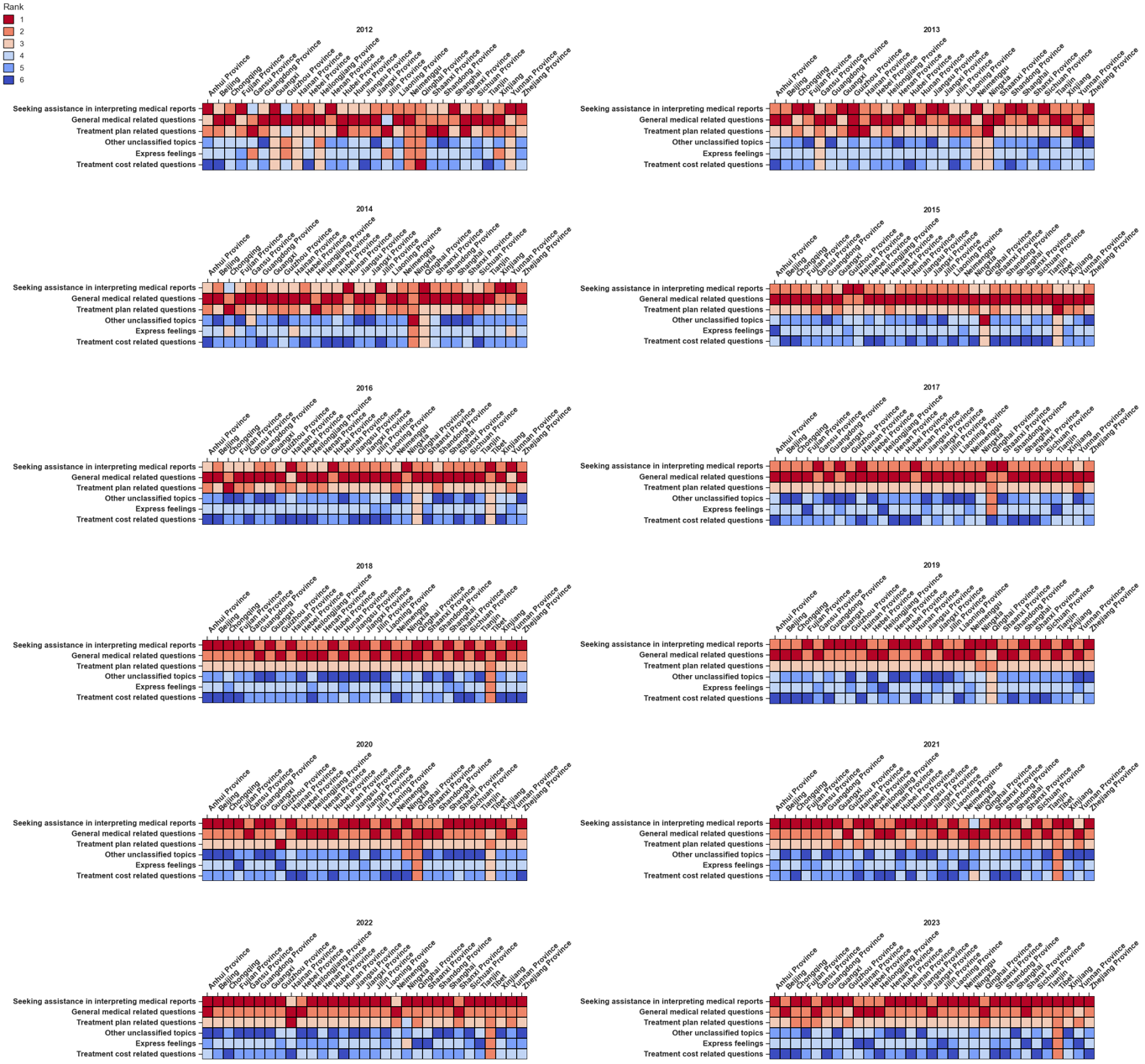

Supplement: Multimedia Appendix 1 [file jmir_v27i1e80497_app1.docx]
